# Decreased Physical Activity during Pregnancy Is Associated with Excessive Gestational Weight Gain

**DOI:** 10.3390/ijerph182312597

**Published:** 2021-11-29

**Authors:** Jia-Jing Sun, Li-Yin Chien

**Affiliations:** 1 School of Nursing, College of Medicine, National Taiwan University, Taipei 112304, Taiwan; c22881@gmail.com; 2 Department of Nursing, Heping Fuyou Branch of Taipei City Hospital, Taipei 112304, Taiwan; 3 Institute of Community Health Care, College of Nursing, National Yang Ming Chiao Tung University, Yang-Ming Campus, Taipei 112304, Taiwan

**Keywords:** pregnancy, physical activity, gestational weight gain, obesity, maternal

## Abstract

The majority of pregnant women in Taiwan are not considered physically active. During pregnancy, many women decrease their physical activity levels when compared to pre-pregnancy. The purpose of this study was to examine the association between decreased physical activity from pre-pregnancy to pregnancy and excessive gestational weight gain (GWG). This study applied a prospective panel design. Recruitment was conducted at six medical facilities in Taiwan and lasted from August 2016 to April 2017. Physical activity levels were determined both before and during pregnancy using the International Physical Activity Questionnaire—Short Form, with data subsequently being transformed into METs-min/week. Excessive GWG was determined based on the body mass index (BMI) specific GWG range. We recruited 747 pregnant women in their second trimester and followed them through to one-month postpartum. About 40% of participants (41.2%) exhibited excessive GWG. Physical activity decreased from an average of 2261 (SD = 3999) to 1252 (SD = 2258) METs-min/week from pre-pregnancy to pregnancy (*p* < 0.0001). Controlling for age and pre-pregnancy BMI, a logistic regression model revealed that a decline in physical activity of > 4000 METs-min/week from pre-pregnancy to pregnancy was associated with an increased risk for excessive GWG (OR = 2.83, 95% CI: 1.27–4.43). A substantial decrease in physical activity from pre-pregnancy to pregnancy was a risk factor for excessive GWG. Although most women decreased their physical activity during pregnancy, only those pregnant women who were physically active pre-pregnancy could show the kind of large decrease that resulted in excessive GWG. Health professionals should continue to develop strategies for counteracting the problematic trend of decreasing PA during pregnancy among low-risk pregnant women.

## 1. Introduction

Estimates have shown that there were approximately 38.9 million overweight pregnant women throughout the world in 2014, with 14.6 million falling into the category of obesity [1]. Although there is a lack of recent data on the global burden of overweightness and obesity among pregnant women, estimates suggest that 20% of women will be obese by 2025 [2]. In this context, maternal obesity has become an increasing area of concern [2]. Looking specifically at Taiwan, a national breastfeeding survey (2012–2016) showed that the proportions of women with overweightness and obesity pre-pregnancy were 10.6% and 5.9%, respectively [3]. This is a notable issue, as women who are overweight or obese at conception are known to have increased risk of excessive gestational weight gain (GWG) and poor pregnancy outcomes [4]. 

Evidence also suggests there is an association between excessive GWG and future overweightness/obesity in the offspring [5,6]. Further, mothers with excessive GWG are at higher risk for preterm birth, cesarean delivery [7], and postpartum weight retention [8]. In response to the obesity epidemic in the Unites States, the American Institute of Medicine (IOM) defined optimal ranges of total GWG for each category of the pre-pregnancy body-mass index (BMI), as follows: 12.5–18 kg for underweight, 11.5–16 kg for normal weight, 7–11.5 kg for overweight, and 5–9 kg for obese women [9]. Weight gains below and beyond the recommended amounts are termed inadequate and excessive GWG, respectively. About 20% and 48% of women in the United States experience inadequate and excessive GWG, respectively [10]. The IOM criteria for GWG based on local cut-off for BMI (18.5, 24, and 27 kg/m^2^ for underweight, normal, overweight, and obese, respectively) are adopted by the Taiwan Health Promotion Administration [11]. A national survey of postpartum women in Taiwan reported that the proportion of women with inadequate and excessive GWG was 41.5% and 22.4%, respectively [12]. Most women actually gain amounts of weight that fall outside the recommendations.

A growing number of studies have examined the efficacy of physical activity (PA) interventions during pregnancy. In this regard, a Cochrane review found no evidence to recommend or discourage exercise during pregnancy in view of its effect on maternal and infant health outcomes [13]. Nonetheless, exercise is often advocated during pregnancy due to its overall health benefits, particularly in preventing chronic diseases and unhealthy weight gain. Furthermore, women with low-risk pregnancies should be able to engage in high-intensity exercise programs that include jogging and aerobic activities, which also help with weight management [14,15,16,17]. In short, research has demonstrated that exercise during pregnancy does not harm the fetus, but can provide benefits for both the mother and baby [14,16]. On the other hand, pregnant women with low PA may be two to three times more likely to experience excessive GWG [7].

The guidelines suggesting that healthy women who are not already highly active or engaging in vigorous activity should spend at least 150 min per week walking or participating in moderate-intensity aerobic activities, while women who already engage in vigorous aerobics or large amounts of activity can continue doing so during pregnancy [14,15,16]. On the other hand, certain aspects of traditional Chinese culture suggest that PA can “disturb the fetus” meaning it should be avoided to prevent miscarriage-like symptoms such as abdominal pain and/or vaginal bleeding [18]. One study found that the median active PA time decreased from 80 min/week (IQR = 0–240 min/week) during pre-pregnancy to 0 min/week (IQR = 0–60 min/week) during both mid- and late pregnancy, with results also showing low overall active PA levels during pregnancy and significant drops in active PA times between pre- and during pregnancy [19].

Previous studies have suggested that regular exercise during pregnancy can reduce GWG [20,21,22], while meta-analyses also concluded that engagement in structured moderate physical exercise programs during pregnancy decreases the risk of gestational diabetes mellitus and diminishes maternal weight gain [21,23]. Nonetheless, a few studies have found that PA (or exercise) was not significantly associated with weight gain during pregnancy [24,25,26]. There was generally a lack of consideration of PA level before pregnancy and the drop in PA level during pregnancy. The literature thus offers no clear or consistent associations between PA and excessive GWG during pregnancy, especially in observational studies. While this may partially be due to the generally high prevalence of low PA during pregnancy, we are therefore not aware of any studies showing that reduced PA during pregnancy is directly associated with GWG. Based on the above and beginning with the hypothesis that excessive GWG is related to decreased PA during pregnancy, this study examined the crude and adjusted associations between decreased PA from pre-pregnancy to pregnancy and excessive GWG. 

## 2. Methods

### 2.1. Design

This study applied a prospective panel design in which women were recruited during their second trimester of pregnancy (gestational weeks 14–27). All participants were evaluated during their second and third trimesters and at one-month postpartum using structured questionnaires. 

### 2.2. Participants and Setting

Participants were recruited from four hospitals and two clinics in Taipei, Taiwan, and the study lasted from August 2016 to April 2017. Ethical approval for this study was obtained from 4 institutional review boards (Far Eastern Memorial Hospital: 105073-F; Taiwan Adventist Hospital: 105-E−03; Taipei City Hospital: TEHIRB−10505103; Buddhist Tzu Chi General Hospital—Taipei Branch: 05-M01–005). This included adult pregnant women who were receiving prenatal care at their respective locations of recruitment. None had pre-existing conditions or experienced complications during early pregnancy (e.g., habitual abortion, diabetes, high blood pressure, and hyperthyroidism). All pregnancies were considered stable. Participants were required to communicate in Mandarin Chinese. After receiving explanations of the study’s purpose, those who agreed to participate signed consent forms and provided research personnel with their contact details, including email addresses, phone numbers, and postal addresses. A total of 985 women met the inclusion criteria, with 800 of those agreeing to participate in this study. Finally, the analysis included 747 women with complete records (successfully followed throughout 1 month postpartum), including information on PA levels and weight. While some were excluded due to missing information (*n* = 53), they did not significantly differ from those with complete information (*n* = 747) in regard to age, education level, work status, and number of pregnancies.

### 2.3. Sample Size Considerations

In the logistic regression model, when the event probability was set at 0.35, the odds ratio was 1.85, and the two-tailed alpha was 0.05, the required sample size to yield a power of 0.80 was 528, based on the sample size calculation using G-Power [27]. Consequently, we considered that the sample size of the study was adequate.

### 2.4. Measures

Study variables included sociodemographic factors (age, work status, education level, and marital status), PA levels, pre-pregnancy BMI, GWG, and parity. Sociodemographic factors, parity, BMI, and pre-pregnancy PA were collected during the second trimester of pregnancy, while PA levels during pregnancy were collected during both the second and third trimesters of pregnancy. 

Physical activity levels were determined using the Taiwanese version of the International Physical Activity Questionnaire—Short Form (IPAQ-SF) [28]. This instrument was used to collect self-reported data on the frequency and duration of walking, moderate-intensity, and vigorous-intensity PA over the preceding seven-day period. A previous study reported that the total PA recorded by IPAQ-SF was acceptably reliable across three times (on days 1, 8, and 11) with an intra-class correlation coefficient (ICC) of 0.79 among Chinese adults in Hong Kong [29]. There were no significant differences in average PA between IPAQ-SF and PA-log, demonstrating acceptable concurrent validity. IPAQ-SF had good test–retest reliability in measuring time spent in moderate PA, vigorous PA, and moderate and vigorous PA (ICC ranged from 0.81–0.84) among pregnant women. However, concurrent validity between IPAQ-SF and an objective PA measure, SenseWear Armband, in measuring time spent in moderate PA, vigorous PA, and moderate and vigorous PA was low to fair (correlation coefficient 0.08–0.39). The low to fair concurrent validity between IPAQ-SF and objective measures were in line with other self-reported PA questionnaires [30].

We followed IPAQ guidelines stating that only 10 or more min of activity should be counted [31]. We then multiplied the metabolic equivalent values for the specific types of PA (e.g., walking = 3.3, moderate activity = 4, vigorous activity = 8) based on the number of min of the activity completed over one week to yield the METs-min/week measurements [32]. A previous study showed that moderate to vigorous PA did not differ between participants in their second and third trimesters of pregnancy [19]. Following this, we used the average METs-min during the second and third trimesters of pregnancy to represent PA levels during pregnancy. The Spearman correlation coefficient of total PA between the second and third trimester was 0.28 (*p* < 0.001) in this study. Pre-pregnancy PA was enquired in the second trimester and the participants were asked to answer their ordinary PA in a typical week before conception based on their recall. 

Maternal weight was obtained from each participant’s Mother’s Handbook or hospital records, while pre-pregnancy BMI was calculated in kg/m² based on maternal pre-pregnancy weight and height in the medical records. We categorized pre-pregnancy BMI using the Taiwanese cutoffs of 18.5, 24, and 27 kg/m^2^ for underweight, overweight, and obesity, respectively [11]. Total GWG was calculated by subtracting the pre-pregnancy weight from the weight at delivery, then categorized as excessive or non-excessive if above or below/within the BMI-specific weight gain ranges established by the Institute of Medicine, respectively [33].

### 2.5. Data Analysis

Statistical analyses were conducted using SPSS for Windows Version 24.0 (IBM Corp, Armonk, NY, USA). Participant characteristics were presented using frequencies and percentages for categorical variables, while means and standard deviations (SDs) were used for continuous variables. The crude associations between excessive GWG and PA or other characteristics were examined using the t-test or chi-squared test. The IPAQ group defined 601–4000 METs-min/week as moderate PA and >4000 METs-min/week as high PA [34]. Given those, PA and decline in PA was divided into <600, 600–4000, and >4000 METs-min/week in the analysis. The logistic regression model was used to examine the net association between PA and excessive GWG. We first fit the logistic regression model by entering all variables, then dropped the least significant variable from the model one at a time. This was repeated until all variables in the model were statistically significant, which was determined based on 2-sided *p* values < 0.05. Adjusted odds ratios (aOR) and 95% confidence intervals (95% CIs) were computed for the logistic model results. 

## 3. Results

### 3.1. Characteristics of the Participants

Table 1 shows the characteristics of the 747 analyzed study participants. The mean age was 33.1 ± 4.4 years (ranging from 20 to 44). As shown, more than 70% (73.2%) had university-level educations or higher, and the majority were married (97.2%). More than half were primiparous (55%). More than one-fifth were overweight or obese before pregnancy (12.6% overweight and 8.4% obese). 

### 3.2. Physical Activity Levels before and during Pregnancy

Before pregnancy, 43.8% and 15.4% of participants had PA levels of 601–4000 and >4000 METs-min, respectively. About 60% revealed declines in PA levels during pregnancy when compared to pre-pregnancy levels; declines in PA (METs) were measured at 21.4%, 29.9%, and 7.8% for decreases of 1–600, 601–4000, and >4000 METs-min, respectively (Table 1).

Table 2 shows the proportions of participants who met the common recommended PA guidelines before and during pregnancy. Mean PA levels decreased from 2261 ± 3999 before pregnancy to 1252 ± 2258 METs-min/week during pregnancy (*p* < 0.001). The proportion of women who reported ≥600 METs-min/week PA decreased from 59.3% before pregnancy to 44.8% during pregnancy (*p* < 0.001). In terms of the type of PA, the decline among the proportion meeting vigorous PA ≥75 min/week was more than that of those meeting moderate PA ≥150 min/week (21.6% to 4.6% for vigorous; 20.9% to 10.8% for moderate; both *p* < 0.001). Walking is the most common type of PA among pregnant women in Taiwan, and is also the type recommended by the Mother’s Handbook; the decline in walking ≥150 min/week appeared to be less pronounced (44.0% to 40.2%, *p* < 0.001). 

The mean PA level was compared before and during pregnancy stratified by whether PA guidelines were met (Table 2). The mean METs-min/week appeared to be higher pre-pregnancy compared to during pregnancy (all *p* < 0.001) except for one. For those whose PA was <600 METs-min/week, their mean METs increased from 170 (before pregnancy) to 438 METs-min/week (during pregnancy; *p* = 0.03). 

Declines in PA levels during pregnancy differed significantly by pre-pregnancy PA level (Table 3). Participants with higher pre-pregnancy PA levels showed greater declines during pregnancy than those with lower PA levels. For each pre-pregnancy PA level, about half of participants experienced substantial decreases during pregnancy when compared to their pre-pregnancy levels (50.4%, 54.4%, and 47.7% of those with >4000, 601–4000, and 1–600 METs-min before pregnancy had decreases of >4000, 601–4000, and 1–600 METs-min during pregnancy, respectively). For participants who were totally inactive before pregnancy (METs = 0), PA levels actually increased during pregnancy, although this was <600 METs-min/week.

### 3.3. Crude Analysis of Factors Associated with Excessive GWG

In this study, 41.2% (*n* = 308) of participants experienced excessive GWG. Table 1 shows the crude associations between participant characteristics and excessive GWG. Of the study variables, pre-pregnancy BMI, pre-pregnancy PA levels, and declined PA during pregnancy were significantly related to excessive GWG. Participants with excessive GWG were significantly more likely to be overweight (23.1% versus 5.2%) and obese (14% versus 4.6%) before pregnancy. Those with excessive GWG showed both a higher mean pre-pregnancy PA level (2881 ± 4868 versus 1826 ± 3188 METs-min, *p* = 0.001) and greater mean decline in PA during pregnancy (1326 ± 3142 versus 785 ± 2657, *p* = 0.014). Using categorized PA levels, 20.8% of participants with excessive GWG were found to have pre-pregnancy PA levels >4000 METS-min, compared to 11.6% of those without excessive GWG. About 11.7% of participants with excessive GWG showed declines >4000 METs-min during pregnancy, compared to 5% of those without excessive GWG. Other variables were not significantly related to excessive GWG.

### 3.4. Multivariate Logistic Regression Model on Factors Associated with Excessive GWG

Table 4 shows the multivariate regression results. Since pre-pregnancy PA was highly related to declined PA during pregnancy, we decided to use declined PA during pregnancy in the modeling. Further, older age was associated with a decreased odds for excessive GWG. Compared to participants who were younger than 30 years of age, those aged ≥40 and 30–39 were less likely to have excessive GWG (OR = 0.37, 95% CI: 0.16–0.86 for ≥ 40; OR = 0.66, 95% CI: 044–0.99 for 30–39). Pre-pregnancy BMI was positively associated with excessive GWG in that the OR for excessive GWG increased as body size increased from normal to overweight/obese comparing to participants who were underweight before pregnancy (ORs = 3.11, 17.29, and 12.71 for normal weight, overweight, and obesity, respectively). After adjusting for age and pre-pregnancy BMI, participants with PA declines >4000 METs-min during pregnancy were 2.38 (95% CI: 1.27–4.43) times more likely to have excessive GWG than those whose PA levels did not decline from pre-pregnancy to pregnancy. Other levels of decline (1–600, 601–4000 METS-min) were not significantly associated with excessive GWG (though OR estimates for these categories were 1.18, and 1.13, respectively). 

## 4. Discussion

This study found that declines in PA levels >4000 METs-min/week from pre-pregnancy to pregnancy were positively associated with excessive GWG. While about 60% of participants decreased their PA levels during pregnancy, only those with high declines (>4000 METs-min/week) showed significant associations with excessive GWG. This finding may not be surprising, since GWG is influenced by many factors other than PA [35]; thus, only substantial declines in PA could retain statistical significance. Further, individuals with reduced levels of PA usually do not exhibit compensatory reductions in energy intake [36,37]. For that reason, a decrease in energy expenditure due to inactivity leads to a positive energy balance, thereby resulting in weight gain [38]. Contrary to previous observational studies, this study found that substantial declines in PA (rather than PA during pregnancy) was associated with excessive GWG. The lack of a significant association between PA and GWG demonstrated by previous observational studies may be due to the fact that PA levels are generally low among pregnant women [24,39]. The recommendations established by the ACOG, CDC, and ACSM assert that healthy women who engage in substantial amounts of PA can continue doing so during pregnancy unless there is a medical reason [16]. Based on the recommendations and our results, we suggest that low-risk pregnant women who are physically active before pregnancy should remain so during pregnancy in order to avoid excessive GWG.

There was a significant decline in the mean PA level from pre-pregnancy to pregnancy (2261 (SD 3999) to 1252 (SD 2258) METs-min/week, respectively). There were significant decreases in several types of PA during pregnancy, including vigorous PA, moderate-intensity PA, and even walking; based on proportions, however, declines were highest for vigorous (−17%), followed by moderate-intensity (−10.1%) and walking (−3.8%). These results are consistent with a previous study showing that, compared to pre-pregnancy levels, pregnant women experienced significant decreases in total PA levels during their second and third trimesters, including both vigorous and moderate-intensity activity types as well as walking [40]. Reasons for these declines may include self-identified physical limitations and restrictions, a lack of resources, decreased energy, and less time for exercise [41]. Further study is needed to examine reasons for decline in PA levels among pregnant women and to compare whether the reasons differed by their pre-pregnancy PA level and BMI group. Due to the overall benefits, pregnant women need exercise programs that are specifically designed around those barriers. In this regard, special attention should be given to help sustain pre-pregnancy PA levels, especially among women who were considered physically active prior to pregnancy.

Interestingly, we also found that about 40% of participants increased their PA levels during pregnancy, with those who were previously inactive being more likely to do so (pre-pregnancy 0 METs: 100%; ≦600 METs: 52.3% versus 601–4000 METs: 19.6%; >4000 METs: 8.7%; Table 3). Increased PA was mostly found in walking during the third trimester. A previous study found that participating women believed exercise helped them stay in shape and prepare for labor/delivery [42]. This study’s results also suggest that the majority of women who are physically inactive before pregnancy may thus be motivated to increase their PA levels while pregnant. Since walking is widely accepted as a beneficial activity among pregnant women, this should be a good way to increase PA levels among women who were not physically active prior to pregnancy. A meta-analysis showed that exercise frequency of three times per week and exercise duration of 30 to 45 min each time can reduce maternal GWG for pregnant women [23]. However, what type and amount of exercise would most benefit pregnant women, or pregnant women with different BMIs or pre-pregnancy PA, remains to be explored by future studies. 

More than 40% of this study’s participants experienced excessive GWG, which supports previous findings [7]. This shows that emphasis should still be placed on appropriate weight gain during pregnancy. Further, more than 20% of participants were overweight/obese before pregnancy, which was also associated with excessive GWG. This also supports previous findings [4]. Health professionals should thus advise pregnant women on healthy GWG during pregnancy, with special attention given to those who are already overweight/obese. 

In this study, a younger maternal age (<30 years) was associated with an increased risk of excessive GWG. The finding may be due to the fact that older women may be more concerned with appropriate GWG and smooth pregnancy experiences; as a result, they place greater emphasis on controlling weight gain during pregnancy. However, there is still controversy about whether this factor is generally associated with an increased or decreased risk for excessive GWG [43,44]. Regardless, the association between maternal age and excessive GWG requires additional research.

### Limitations

The study women were from six clinics in a metropolitan area in northern Taiwan, posing concerns to the generalizability of the results. The study participants appeared to be older and more educated than the national data (mean age 32.12 and 58% with an educational level of university or higher in 2019 [45]). Although pre-pregnancy weights and heights were taken from medical records, pre-pregnancy weights were usually self-reported. As such, under- and over-reporting were possible issues. Physical activity was also self-reported; however, previous studies have supported the validity of the IPAQ-SF, which was used in this study [29]. Specifically, the IPAQ-SF enquires about PA during the preceding seven-day period; we averaged the two measures taken during the second and third trimesters to represent PA during pregnancy, since the two measures appeared to be similar. There was a lack of consideration of PA during the first trimester or of potential weekly differences in PA. Pre-pregnancy PA was self-reported in the second trimester and recall bias as well as the accuracy of the report may be a concern. Future studies should collect PA more often and include PA levels collected before pregnancy and during the first trimester in order to gain a more complete picture. In addition, an objective measure of PA such as an accelerometer could be used to further examine the validity of the IPAQ-SF and capture objective PA levels. A smaller effect may not be detected. We did not collect information on the total dietary calorie intake due to the complexity of the measure. Such unmeasured confounders could have influenced our results and should be considered in future studies.

## 5. Conclusions

A decrease in physical activity >4000 METs-min/week from pre-pregnancy to pregnancy was found to be a risk factor for excessive GWG. Women with substantial declines in PA from pre-pregnancy to pregnancy are usually those who were physically active prior to becoming pregnant. Health professionals should continue to develop strategies for counteracting the problematic trend of decreasing PA during pregnancy among low-risk pregnant women.

## Figures and Tables

**Table 1:** Participant Characteristics by Excessive Gestational Weight Gain (*n* = 747).

			Excessive Gestational Weight Gain		
Sample Characteristics		Total *n* (%) *N* = 747	No	Yes	*p*-Value
			*n* (%) *n* = 439	*n* (%) *n* = 308	
Age (years)					0.255
	20–29	146 (19.5)	80 (18.2)	66 (21.4)	
	30–39	562 (75.2)	332 (75.6)	230 (74.7)	
	≥40	39 (5.2)	27 (6.2)	12 (3.9)	
Education					0.672
	High school or less	126 (16.9)	71 (16.2)	55 (17.9)	
	College/Vocational school	74 (9.9)	45 (10.3)	29 (9.4)	
	University	435 (58.2)	252 (57.4)	183 (59.4)	
	Postgraduate	112 (15.0)	71 (16.2)	41 (13.3)	
Work status					0.492
	Employed	552 (73.9)	325 (74)	227 (73.7)	
	Unemployed	195 (26.1)	114 (26.0)	81 (26.3)	
Currently married					0.523
	Yes	726 (97.2)	427 (97.3)	299 (97.1)	
	No	21 (2.8)	12 (2.7)	9 (2.9)	
BMI (kg/m^2^)					<0.0001
	<18.5 (underweight)	110 (14.7)	93 (21.2)	17 (5.5)	
	18.5–24.9 (normal)	480 (64.3)	303 (69.0)	177 (57.5)	
	25–26.9 (overweight)	94 (12.6)	23 (5.2)	71 (23.1)	
	≥27 (obese)	63 (8.4)	20 (4.6)	43 (14.0)	
Number of pregnancies					0.08
	1	411 (55.0)	227 (51.7)	184 (59.7)	
	2	282 (37.8)	176 (40.1)	106 (34.4)	
	≥3	54 (7.2)	36 (8.2)	18 (5.8)	
PA before pregnancy (METs-min/week)					0.005 **
	0	152 (20.3)	99 (22.6)	53 (17.2)	
	1–600	153 (20.5)	94 (21.4)	59 (19.2)	
	601–4000	327 (43.8)	195 (44.4)	132 (42.9)	
	>4000	115 (15.4)	51 (11.6)	64 (20.8)	
Declines in PA during pregnancy than before (METs-min/week)					
	No decline	306 (40.9)	190 (43.3)	116 (37.7)	0.008 *
	1–600	160 (21.4)	94 (21.4)	66 (21.4)	
	601–4000	223 (29.9)	133 (30.3)	90 (29.2)	
	>4000	58 (7.8)	22 (5.0)	36 (11.7)	

Abbreviations: BMI = body mass index; SD = standard deviation; PA = physical activity. *p*-values were calculated using the chi-squared test or *t*-test * *p* < 0.05, ** *p* < 0.01.

**Table 2:** Physical Activity Before and During Pregnancy (*N* = 747).

Physical Activity		Pre-Pregnancy		During Pregnancy	Mean MET (SD)
		*n* (%)	Mean METs *n* (%) (SD)		
Total physical activity			2261 (3999)		1252 (2258)
Vigorous (≥75min/wk)					
	No	586 (78.4)	1618 (2985)	713 (95.4)	1150 (2071)
	Yes	161 (21.6)	4603 (5913)	34 (4.6)	1625 (2815)
Moderate (≥150min/wk)					
	No	591 (79.1)	1448 (2448)	666 (89.2)	947 (1702)
	Yes	156 (20.9)	5340 (6488)	81 (10.8)	2408 (3438)
Walking (≥150min/wk)					
	No	418 (56.0)	857 (2535)	447 (59.8)	690 (1504)
	Yes	329 (44.0)	4045 (4743)	300 (40.2)	1967 (2794)
≥ 600METs-min/wk					
	No	304 (40.7)	170 (201)	412 (55.2)	438 (679)
	Yes	443 (59.3)	3696 (4679)	335 (44.8)	1811 (2743)

The comparisons of PA level at pre-pregnancy to pregnancy were performed using the chi-squared test or Student’s *t*-test. All *p* were < 0.001 except for the mean METs between pre- and pregnancy at “Walking (≥150 min/wk) No,” where *p* = 0.03. Abbreviations: METs = METs-min/week; min = min; wk = week.

**Table 3:** Declines in Physical Activity from Pre-pregnancy to Pregnancy Based on Pre-Pregnancy Levels (*n* = 747).

Decline in Physical Activity during Pregnancy	Physical Activity Before Pregnancy ^a^				
	0	1–600	601–4000	≥4000	
	*n* (%)	*n* (%)	*n* (%)	*n* (%)	Total
No decline	152 (100)	80 (52.3)	61 (19.6)	10 (8.7)	306 (41)
1–600	0	73 (47.7)	85 (26)	2 (1.7)	160 (21.4)
601–4000	0	0	178 (54.4)	45 (39.1)	223 (29.9)
>4000	0	0	0	58 (50.4)	58 (7.8)
Total	152 (20.3)	153 (20.5)	327 (43.8)	115 (15.4)	747

*p*-values obtained via Fisher’s exact test for frequency comparisons. *p* < 0.0001. ^a^ METs-min/week.

**Table 4:** Logistic Regression Model for Factors Associated with Excessive Gestational Weight Gain (*n* = 747).

		OR ^a^	95% CI	*p*-Value
Age (years)				
	20–29	1		
	30–39	0.66	(0.44–0.99)	0.045 *
	≥40	0.37	(0.16–0.86)	0.021 *
Pre-pregnancy BMI				
	Underweight	1		
	Normal	3.11	(1.78–5.41)	<0.0001 ***
	Overweight	17.29	(8.51–35.15)	<0.0001 ***
	Obese	12.71	(6.00–26.89)	<0.0001 ***
Declines in physical activity during pregnancy (METs)				
	No decline	1		
	1–600	1.18	(0.77–1.80)	0.441
	601–4000	1.13	(0.77–1.66)	0.519
	>4000	2.38	(1.27–4.43)	0.006 **

^a^. OR, odds ratio. ORs were adjusted for other variables in the model. Abbreviations: BMI = body mass index; CI = confidence interval; *p* < 0.05 *, *p* < 0.01 **, *p* < 0.001 ***.

## Data Availability

The datasets generated and/or analyzed during the current study are not publicly available due to confidentially reasons, but are available from the corresponding author on reasonable request.
